# Abdominal Tuberculosis in Children: A Case Series of Five Patients

**DOI:** 10.3390/microorganisms11030730

**Published:** 2023-03-12

**Authors:** Laura Lancella, Luciana Abate, Laura Cursi, Giulia Chiopris, Laura Nicoletti, Nicola Principi, Alberto Villani, Susanna Esposito

**Affiliations:** 1 Academic Department of Pediatrics (DPUO), Infectious Disease Unit, Bambino Gesù Children’s Hospital, IRCCS, 00165 Rome, Italy; 2 Pediatric Clinic, Department of Medicine and Surgery, University of Parma, 43126 Parma, Italy; 3 Università degli Studi di Milano, 20122 Milan, Italy

**Keywords:** abdominal tuberculosis, anti-infective therapy, *Mycobacterium tuberculosis*, pediatric infectious diseases, TB, tuberculosis

## Abstract

Tuberculosis remains (TB) to be one of the most common causes of child morbidity and mortality. Abdominal TB is not frequently diagnosed and, although its incidence is not definitively established, there are data that seem to indicate that it accounts for approximately 1–3% of all pediatric TB cases and for no more than 10% of those with extrapulmonary manifestations. It seems, however, that abdominal TB is significantly more common than usually thought as signs and symptoms are non-specific and may mimic other diseases. The delayed or wrong diagnosis of pediatric abdominal TB can have dramatic consequences as they can lead to untreated TB with miliary dissemination, unnecessary surgery, or dangerous drug therapies. This report describes five cases of abdominal TB diagnosed among 216 pediatric patients admitted for TB in Italy from 2011 to 2021. Our cases evidence that abdominal TB is a complex and potentially very severe disease that, when not appropriately diagnosed, may be associated with severe complications and prolonged anti-TB therapy. Discussion among specialists is crucial to achieve an early diagnosis and to promptly start the anti-TB treatment. Further studies are needed to clarify the appropriate duration of therapy as well as management of MDR abdominal TB cases.

## 1. Background

Tuberculosis remains (TB) to be one of the most common causes of child morbidity and mortality. The World Health Organization (WHO) has estimated that in 2021, more than 550,000 children fell ill with TB and that TB was the main cause of death in 224,000 children [1]. Although most pediatric TB cases and deaths have occurred in the developing world, particularly in South East Asia, Africa and the Western Pacific, a nonmarginal number of cases has been diagnosed in industrialized countries, particularly in Europe [1]. A strong contribution to increasing the importance of TB in pediatrics also has had evidence that even in countries with the lowest incidence of TB treatment of the disease can be significantly complicated by the emergence of multidrug-resistant (MDR) *M. tuberculosis* strains [2]. This explains why research on pediatric TB is of interest to many health authorities. To assure the best approach to children with TB, an improvement in the monitoring of TB epidemiology, clinical manifestations and response to therapy is strongly suggested [3]. Particular attention should be paid to extrapulmonary manifestations. Regions with low TB prevalence account for about 20% of all the pediatric TB cases, and because of their nonspecific clinical presentation, they can be diagnosed and treated very late and have a very unfavorable evolution [4]. Lymph nodes, meninges, and the musculoskeletal system are the most common sites of extrapulmonary TB. Abdominal TB is less frequently diagnosed, and although its incidence is not definitively established, there are data that seem to indicate that it accounts for approximately 1–3% of all pediatric TB cases and for no more than 10% of those with extrapulmonary manifestations [5,6]. It seems, however, very likely that abdominal TB is significantly more common than what was usually thought. It can present with very different signs and symptoms because TB can affect the abdominal lymph nodes, intestine, peritoneum, liver, spleen, and kidney at the same time or separately [7,8,9,10,11]. These different clinical manifestations frequently have an insidious onset without constitutional signs and symptoms [12], and can mimic manifestations of other abdominal diseases, mainly intestinal perforation, appendicitis, and Crohn’s disease [13]. All these factors make it possible that many abdominal TB cases escape precise identification, especially in the first phases of the disease. On the other hand, the risk that a relevant part of abdominal TB may be underdiagnosed seems confirmed by the evidence that 37.5% of children admitted to the hospital and later died for lung TB had, at autopsy, signs of a previously undiagnosed intestinal infection [14].

The delayed or wrong diagnosis of pediatric abdominal TB can have dramatic consequences as they can lead to untreated TB with miliary dissemination, unnecessary surgery, or dangerous drug therapies. This report describes five cases of abdominal TB in children that can be helpful to improve the diagnostic and therapeutic approach to this potentially very severe disease.

## 2. Case Presentation

We collected data from 216 pediatric patients admitted for TB in Bambino Gesù Children’s Hospital-Infectious disease Unit in Rome from 2011 to 2021. Five cases of abdominal tuberculosis from this cohort have been reported and described below.

**Case 1**. A female 12 years and 3 months old, from India, received the BCG vaccine at birth. Index case was not known. She presented with acute abdomen, complaining of lower quadrant colic abdominal pain for four days and vomiting. Initial differential diagnosis included sub-occlusion and acute appendicitis. Abdominal X-ray and ultrasound were performed, confirming occlusion/sub-occlusion due to the presence of air-fluid levels.

The computed tomography (CT) scan showed multiple and non-conglobate lombo-aortic lymphomegalies found in the hepato-gastric ligament, the hepatic ilo, and in the mesentery along the ileocolic vessels as well; adipose tissue in the mesentery appeared mildly hyperdense and nodular. Small bowel loops looked moderately distended by fluid with thickened and edematous walls; a small quantity of free fluid was found in the abdomen.

On day two of admission, exploratory laparotomy was performed, adhesions were detached and ascitic fluid was drained. Surgery highlighted both visceral and parietal peritoneum to be very thickened and fleshy in consistency, with small hard and whitish nodules along the omentum (tuberculoma) and omentum–peritoneal adhesions.

Bowel loops were distended by fluid content, with edematous, extremely friable, and easily bleeding walls; several viscero-visceral adhesions were found, one of which was restricting one ileal loop. The same lesions were affecting the ovaries.

The histological examination described fibro-fatty tissue with epithelioid granulomas and multinucleated giant cells and central calcification but no signs of necrosis.

Considering all the investigations performed, the patient was diagnosed with generalized hematogenous tuberculosis, affecting the spine, soft tissues, the superior and inferior diaphragmatic lymph nodes, lungs, and the mediastinum.

The Mantoux tuberculin skin test (TST) and the Quantiferon test were positive. *M. tuberculosis* was found by the polymerase chain reaction (PCR) test and culture on the sputum, although the acid-fast bacillus smear was negative.

A quadruple anti-TB regimen was given for two months, with isoniazid, rifampicin, levofloxacine and ethambutol; the considered resistance to pyrazinamide was revealed by the antibiogram. Dual therapy with isoniazid and rifampicin (I-R) was extended for one year due to concerns that the 6-month duration may not be adequate to achieve cure and prevent relapse after the end of treatment.

The girl received prophylactic supplementation with vitamin B6 for neuritis prevention and she required one red blood cell transfusion. She developed iatrogenic liver injury with elevations of AST, ALT, and gamma-GT that required a temporary reduction in the antibiotic doses; furthermore, allopurinol (10 mg/kg/day) was needed to control hyperuricemia.

The patient completed her 12-month follow-up after the end of therapy and fully recovered without any other complications.

**Case 2**. A male 15 years and 9 months old, from the Philippines, received the BCG vaccine at birth. Index case was not known. The boy presented with a history of colic abdominal pain, anorexia, low-grade fever, and weight loss of 7 kg in two months. An abdominal X-ray and ultrasound showed distended colonic and jejunal-ileal loops, air-fluid levels, and free fluid, which was compatible with bowel obstruction. The CT scan confirmed this finding, with thickening of the ileal loop walls, mild periportal edema, and multiple mesenteric adenopathies.

A total of 24 h after admission, laparotomy was performed, with deflation of the loops, aspiration of intestinal content, and consequent ileostomy. A manual examination revealed the ileal loops to be bulky, distended, and attached to the pelvi and to the omentum. Moreover, several hard, whitish, not-mobile nodules were found on the mesentery and ileal loops (tuberculoma).

A histopathological analysis of mesentery and of the lymph nodes showed granulomatous inflammation with multinucleated giant cells and caseous necrosis. Peritoneal fluid contained neutrophils, small lymphocytes, macrophages, and a few reactive mesothelial cells. Culture was not performed.

The abdomen was the only site affected by the disease. Quantiferon was positive; TST was not performed. 

Lacking susceptibility testing, the anti-TB therapy was started with isoniazid, rifampicin, pyrazinamide, and ethambutol for two months, followed by I-R for ten months. Supplementation with vitamin B6 and vitamin D was given. The patient required one albumin infusion. He developed liver injury and hyperuricemia.

As a result of the surgery, he developed short-bowel syndrome and still requires the ileostomy: surgical recanalization has been scheduled for the next months. After the discharge, he was admitted again twice for bowel obstruction due to the adhesions.

**Case 3**. A female 10 years and 7 months old from Romania received the BCG vaccine at birth. The index case was the grandmother.

One month before her arrival in Italy, she was admitted in her own country. The initial clinical and radiological suspicion was acute appendicitis due to fever, vomiting, and right-side abdominal pain, so she underwent appendectomy. The Ziehl–Neelsen stain performed on the biopsy of the appendix was positive for acid fast bacilli so she was diagnosed as intestinal TB.

She was taken to our hospital where an abdominal ultrasound detected small ovalar, enlarged mesenteric lymph nodes, mostly in the right iliac fossa, with no other abnormalities affecting the loops and no free fluid. An abdominal CT revealed hepatomegaly and confirmed multiple adenopathies within the mesenteric adipose tissue.

A histopathological analysis conducted in our facility confirmed the finding of chronic granulomatous lymphadenitis with multinucleated giant cells; the intestinal samples were affected as well by active granulomatous transmural inflammation with leukocyte infiltration, superficial ulcers, several eosinophils in the mucosa, and submucosa; additionally, rare granulomas with caseous necrosis were detected.

TB foci were localized in the gut. The Quantiferon was positive; TST and biopsy culture were not performed. The patient received isoniazid, rifampicin, pyrazinamide, and ethambutol for two months, followed by I-R for ten months for fear of relapse after the end of the traditional 6-month therapy. Supplementation with vitamin B6 and vitamin D was given. The girl developed liver disease.

She carried on the follow-up in her home country.

**Case 4**. A female 14 years and 10 months old from Perù; she had not received the BCG vaccine in the past. No index case was recognized.

The disease started with epigastric and right fossa pain, diarrhea, anorexia, and weight loss of approximately 7 kg in the last year.

Abdominal ultrasounds (USs) and CT revealed parietal thickening of abdominal loops and partial loss of wall stratification of the last ileal loop, of the caecum, and ascending colon with several reactive lymphadenopathies.

Colonoscopy was performed because of a suspicion of Crohn disease, showing multiple, non-confluent aphthoid ulcers and micro polyps in the terminal ileum; the caecum and proximal transverse colon looked edematous with linear and confluent ulcers. No stenosis was described. The ileocecal valve, the terminal transverse colon, the descending colon, the sigmoid colon, and the rectum were free from disease.

Both PCR and cultures conducted in the stool sample were positive for *M. tuberculosis*. Histological analysis showed chronic inflammation with rare multinucleated giant cells, decreased crypt density, and crypt distortion in the terminal ileum, caecum, ascending, and transverse colon. Furthermore, a dense neutrophilic, eosinophilic, and plasmacytic infiltrate was found in the lamina propria and in the epitelium; this was associated with cryptic abscess but no epithelioid granuloma.

The Ziehl–Neelsen stain was negative. After paraffin extraction, a high-sensitivity quantitative PCR test was performed, detecting *M. tuberculosis* DNA on the caecum and ascending colon mucosa. The point of insertion IS6110 was used as a genetic marker and 3030/10^6^ cell copies of DNA were found. The girl was affected by bilateral cavitating pulmonary tuberculosis and *M. tuberculosis* was isolated from the sputum as well.

The Quantiferon test and TST showed positive results.

Anti-TB therapy based on isoniazid, rifampicin, pyrazinamide, and ethambutol was carried on for two months, followed by I-R for ten months for fear of relapse after the end of the traditional 6-month therapy. Supplementary vitamin B6 and vitamin D were given. Clinical improvement was achieved with a course of oral prednisolone for 8 weeks associated with a lactose-free diet and a special milk formula for Crohn’s disease (Modulen). In this case, the girl developed lower limb paraesthesia that responded to ibuprofen and to vitamin B6 supplementation.

During the 12-month follow-up after the end of therapy, no other complications have been reported.

**Case 5**. A male 11 years and 3 months old from Pakistan received the BCG vaccine at birth. He was affected by congenital bilateral sensorineural hearing loss and pigmentary mosaicism. No index case was recognized.

The starting symptoms included progressive fatigue, chest pain, epistaxis, and diarrhea.

Abdominal US revealed multiple mesenteric and para-aortic lymphadenopathies complicated with calcification and colliquative necrosis, and thickening of the last ileal loop and of the appendix. A CT scan confirmed the severe parietal thickening involving the last ileal loop, the ileo-caecal valve, the caecum, the appendix, and the proximal ascending colon with concomitant dishomogeneity of the peri visceral fatty tissue. The US finding concerning the lymph nodes was confirmed too, especially in the mesentery and along the ileocolic vessels. In this case, the infection was systemic, involving the brain, lungs with cavitary form, mediastinum, neck, and axillary lymph nodes.

The acid-fast bacillus smear, PCR, and culture in the sputum were all positive for *M. tuberculosis*. The TST and Quantiferon were positive.

The standard four-drug regimen was given for two months and completed by I-R for ten months fearing relapse after the end of the traditional 6-month therapy. The patient received vitamin B6 and vitamin D supplementation and she required one red blood cell transfusion.

The patient completed her 12-month follow-up after the end of therapy without any other complications.

Table 1 summarizes characteristics of abdominal TB in these five children.

## 3. Discussion

This paper describes five cases of pediatric abdominal TB, three with secondary involvement and two with primary abdominal TB. Although the number of studied children is too small to allow for conclusions on the true incidence of primary abdominal TB, it is enough to underline the importance of an accurate evaluation of all the children with abdominal discomfort, especially when laboratory findings indicate TB infection, and they were living in countries with high incidences of TB.

It should be highlighted that in most developing countries, TB is very common, children are immunized with the BCG vaccine at birth, and differentiation of those with active TB and those without infection can preferably be made using T-cell-based interferon-gamma release assays (IGRAs). These tests can also be useful for the identification of patients with TB infection and abdominal TB involvement that could be confused with gastrointestinal diseases of other origins but presenting with similar symptoms. Crohn’s disease was initially considered among the potential illnesses from which one of the patients here reported could be suffering from, but the positive of the IGRA test, allowed, together with medical images, to make the right diagnosis. Moreover, abdominal TB should be considered for any child suffering from prolonged unexplained abdominal problems, even if clinically marginal. It cannot be forgotten that most of the abdominal TB cases present with abdominal pain are eventually associated with abdominal distension and constipation or diarrhea and vomiting, and the signs and symptoms commonly ascribed to TB as fever, weight loss, fatigue, and nocturnal sweating develop later [15]. Abdominal US to evaluate lymph nodes is essential [16,17,18] and, in positive cases, CT may add information not only on lymph nodes and the compression they exert on the adjacent structures, but also on the thickening of the peritoneum or the intestinal wall and the loss of the regular wall stratification of the loops [19,20,21].

The cases of pediatric abdominal TB described in this paper highlight how difficult the identification of the disease can be and how important it can be for an early diagnosis in order to prevent an uncontrolled evolution with development of severe abdominal damage associated with potential long-term effects into adulthood [11,22]. All the reported cases, regardless of the primary or secondary pathogenesis, had significant intestinal, lymph nodal, and peritoneal changes that seem to indicate a long-lasting previously unknown or insufficiently treated disease. This seems to be confirmed by the mean age at diagnosis of these patients. The mean age was 12 years—a value significantly higher than the value of less than 5 years, of which was found in previous studies [23]. On the other hand, observational studies have shown that the majority of pediatric TB diseases are diagnosed in young children that are also more likely to develop disseminated or extrapulmonary TB than older subjects [24,25].

Two different pathogenetic mechanisms of abdominal TB have been described [26]. The first regard the ingestion of mycobacteria. *M. tuberculosis* may reach the intestine through the ingestion of infected sputum coming from the lung of patients with extensive pulmonary cavitation [25]. Similarly, the ingestion of cow milk infected by *Mycobacterium bovis* can be the source of the intestinal infection. In industrialized countries, this last method of abdominal TB development only exceptionally occurs as infected cattle are culled and the cow milk is systematically pasteurized [27]. This cannot be excluded in patients living in developing countries, where these measures are not always put in place. However, this is not the case for the patients here described, at last for the children with detailed microbiological evaluation. The second pathogenetic mechanism is the lymphohematogenous dissemination of *M. tuberculosis* from an infected site, mainly the lung with the secondary involvement of abdominal structures. An early diagnosis of abdominal TB helps to avoid the onset of a dramatic and dangerous acute event. In three of the five cases reported here, surgery was required to remove intestinal obstruction and in one of these cases, because of the intestinal resection, short bowel syndrome was caused, and long-term ileostomy was required. Obstruction is the most common complication of intestinal TB and is mainly due to hyperplastic cecal mural thickening, ileal strictures, and/or adhesions. All these alterations were clearly documented in our patients in whom lymphadenopathy and peritonitis were also evidenced, confirming that although liver, spleen, and kidney involvement is relatively uncommon, the simultaneous damage of intestine, lymph nodes, and peritoneum is frequent [26,27].

The diagnosis of abdominal TB can be strongly suspected on the base of imaging findings and/or laparoscopy/laparotomy findings, but can only have a definitive confirmation when *M. tuberculosis* is detected in abdominal specimens (i.e., ascites, lymph nodes, or peritoneal tissue biopsy during surgery) or, in secondary cases, in the respiratory or gastric secretions through smear microscopy, culture, or molecular biology methods [28]. Moreover, the detection of *M. tuberculosis* is useful to prescribe the most effective therapy as the presence of bacterial resistance to commonly used antibiotics can be evaluated [29]. Unfortunately, abdominal TB is often a paucibacillary disease [15]. Furthermore, smear microscopy is poorly sensitive, whereas culture is time-consuming (3–8 weeks) and the results are frequently negative. In many patients, as shown in case 1 of our series, the detection of *M. tuberculosis* could only be possible using molecular methods [30].

Regarding TB treatment, it is recommended that pediatric patients with abdominal TB receive a four-drug regimen during the intensive treatment phase [31,32]. Although there is not a clear consensus on the duration of therapy, generally, the administration of isoniazid, rifampicin, pyrazinamide, and ethambutol within the first two months (intensive phase) and then isoniazid and rifampicin in the last 4–10 months (i.e., the continuation phase) represents the therapeutic schedule of choice. We treated our patients for an overall period of 12 months due to the complicated clinical manifestations at diagnosis. Ethambutol is included in this regimen because the bacterial load is diagnosed as extremely high and the presence of ethambutol as a companion drug offers increased protection against bacterial resistance. Obviously, when laboratory tests indicate a resistance to first-line anti-TB drugs, adequate modifications must be made [33,34].

## 4. Conclusions

Our cases evidence that abdominal TB is a complex and potentially very severe disease that, when not appropriately diagnosed, may be associated with severe complications and the need for surgical intervention followed by prolonged anti-TB therapy. Discussion among specialists is crucial to achieve an early diagnosis and to promptly start the anti-TB treatment because early treatment is one of the most important positive prognostic factors. Further studies are needed to clarify the appropriate duration of therapy as well as the management of MDR abdominal TB cases.

## Figures and Tables

**Table 1:** Characteristics of abdominal tuberculosis (TB) in five children.

Case	Age	Sex	Origin	BCG Vaccine	Index Case	Underlying Disorders	Symptoms of Onset	Laboratory and Microbiological Examination	Radiological Examination	Histological Examination	Other Localizations	Therapy	Outcome
1	12 (3/12)	F	India	Yes	Not known	No	Lower quadrant colic abdominal pain, vomit	Neutrophilic leukocytosis, rising indexes of inflammation, positive gastric aspirate, positive TST test, positive Quantiferon test	Abdominal X-ray: bowel occlusion/subocclusion (presence of air-fluid levels). Abdominal CT scan: multiple and non-conglobate lombo-aortic lymphomegalies and in hepato-gastric ligament, hepatic ilo and in the mesentery; small bowel loops distented by fluid with thickened and edemayous walls; free fluid in the abdomen.	Fibro-fatty tissue with epithelioid granulomas and multinucleated giant cells with central calcification	Hematogenous tuberculosis, affecting the spine, the soft tissues, the superior and inferior diaphragmatic lymph nodes, the lungs, and the mediastinum.	12 months of therapy Quadruple anti-TB regimen for 2 months (pyrazinamide substituted with levofloxacin for resistance) Dual therapy (I-R) was extended for 1 year	No complications
2	15 (9/12)	M	The Philippines	Yes	Not known	No	Colic abdominal pain, low-grade fever, anorexia, weight loss (7 kg in two months)	Neutrophilic leukocytosis, rising indexes of inflammation, hyp-oalbuminemia, increased fecal calprotectin, TST test not performed, positive Quantiferon test	Abdominal X-ray: bowel occlusion/subocclusion (presence of air-fluid levels). Abdominal ultrasound: distended colonic and jejunal-ileal loops Abdominal CT scan: bowel occlusion with thickening of the ileal loop walls, mild periportal edema and multiple mesenteric adenopathies.	Granulomatous inflammation with multinucleated giant cells and caseous necrosis. Peritoneal fluid contained neutrophils, small lymphocytes, macrophages and few reactive mesothelial cells.	No	12 months of therapy Quadruple anti-TB regimen for 2 months Dual therapy (I-R) was extended for 1 year	Short-bowel syndrome Carrier of ileostomy (awaiting surgical recanalization) Two bowel obstruction due to the adhesions
3	10 (7/12)	F	Romania	Yes	Grand-mother	No	Right-side abdominal pain, fever, vomiting	TST test not performed, positive Quantiferon test	Abdominal ultrasound: small ovalar lymph nodes in mesenteric adipose tissue and in the right iliac fossa. Abdominal CT scan: hepatomegaly and multiple adenopathies within the mesenteric adipose tissue.	Chronic granulomatous lymphadenitis with multinucleated giant cell and granulomatous transmural inflammation and superficial ulcers; rare granulomas with caseous necrosis	No	12 months of therapy Quadruple anti-TB regimen for 2 months Dual therapy (I-R) was extended for 1 year	Lost to follow-up
4	14 (10/12)	F	Perù	No	Not known	No	Epigastric and right-side abdominal pain, diarrhea, anorexia, weight loss (7 kg in last year)	Neutrophilic leukocytosis, rising indexes of inflammation, positive gastric aspirate, hyp-oalbuminemia, increased fecal calprotectin, positive TST test, positive Quantiferon test	Abdominal CT scan: parietal thickening of abdominal loops, partial loss of wall stratification of the last ileal loop, of the caecum and ascending colon with several reactive lymphadenopathies. Colonoscopy: multiple, non-confluent aphthoid ulcers and micro polyps in the terminal ileum; edema of caecum and proximal transverse colon with linear confluent ulcers.	Chronic inflammation and multinucleated giant cells, dense inflammatory lymphoplasmacellular infiltrate, neutrophilic and eosinophilic granulacitic cells in the lamina propria and intraepitheliumwith aspects of cryptitis (rarefied and dysmorphic) and cryptic abscesses.	Bilateral cavitating pulmonary tuberculosis	12 months of therapy Quadruple anti-TB regimen for 2 months Dual therapy (I-R) was extended for 1 year	No complications
5	11 (3/12)	M	Pakistan	yes	not known	Congenital bilateral sensorineural hearing loss and pigmentary mosaicism	Diarrhea, progressive fatigue, chest pain, epistaxis	Rising in indexes of inflammation, positive gastric aspirate, hypo-albuminemia, increased fecal calprotectin, positive TST test, positive Quantiferon test	Abdominal US: multiple mesenteric and para-aortic lymphadenopathies with calcification and colliquative necrosis, thickening of the last ileal loop and of the appendix. Abdominal CT scan: severe parietal thickening of the last ileal loop, the ileo-caecal valve, the caecum, the appendix and the proximal ascending colon with concomitant dishomogeneity of the peri visceral fatty tissue. Multiple lymph nodes in the mesentery and along the ileocolic vessels.	No	Systemic infection involving the brain, lungs with cavitary form, mediastinum, neck, and axillary lymph nodes	12 months of therapy Quadruple anti-TB regimen for 2 months Dual therapy (I-R) was extended for 1 year	No complications

CT, computed tomography; TST, tuberculin skin test; US, ultrasound.

## Data Availability

All the data are included in the manuscript.

## References

[1] World Health Organization Global Tuberculosis. Report 2022. https://www.who.int/teams/global-tuberculosis-programme/tb-reports/global-tuberculosis-report-2022.

[2] Harichander S., Wiafe E., Mensah K.B., Bangalee V., Oosthuizen F. (2022). The incidence of TB and MDR-TB in pediatrics and therapeutic options: A systematic review. Syst. Rev..

[3] Vukugah T.A., Ntoh V.N., Akoku D.A., Leonie S., Jacob A. (2022). Research Questions and Priorities for Pediatric Tuberculosis: A Survey of Published Systematic Reviews and Meta-Analyses. Tuberc. Res. Treat..

[4] Santiago-García B., Blázquez-Gamero D., Baquero-Artigao F., Ruíz-Contreras J., Bellón J.M., Muñoz-Fernández M.A., Mellado-Peña M.J. (2016). Pediatric Extrapulmonary Tuberculosis: Clinical Spectrum, Risk Factors and Diagnostic Challenges in a Low Prevalence Region. Pediatr. Infect. Dis. J..

[5] Basu S., Ganguly S., Chandra P.K., Basu S. (2007). Clinical profile and outcome of abdominal tuberculosis in Indian children. Singap. Med. J..

[6] Sheer T.A., Coyle W.J. (2003). Gastrointestinal tuberculosis. Curr. Gastroenterol. Rep..

[7] Sartoris G., Seddon J.A., Rabie H., Nel E.D., Schaaf H.S. (2020). Abdominal Tuberculosis in Chil-dren: Challenges, Uncertainty, and Confusion. J. Pediatr. Infect. Dis. Soc..

[8] Lin Y.-S., Huang Y.-C., Lin T.-Y. (2010). Abdominal tuberculosis in children: A diagnostic challenge. J. Microbiol. Immunol. Infect..

[9] Tinsa F., Essaddam L., Fitouri Z., Brini I., Douira W., Ben Becher S., Boussetta K., Bousnina S. (2010). Abdominal tuberculosis in children. J. Pediatr. Gastroenterol. Nutr..

[10] Vanhoenacker F.M., De Backer A.I., Op de B.B., Maes M., Van Altena R., Van Beckevoort D., Kersemans P., De Schepper A.M. (2004). Imaging of gastrointestinal and abdominal tuberculosis. Eur. Radiol..

[11] Sartoris G., Seddon J.A., Rabie H., Nel E.D., Losurdo G., Schaaf H.S. (2020). Abdominal Involvement in Children With Bacteriologically Confirmed Tuberculosis: A Five-year Experience From Cape Town, South Africa. Pediatr. Infect. Dis. J..

[12] Al-Fadel S.M., Al-Quorain A., Larbi E., Al-Fawaz I., Taha O., Satti M.B. (1997). Tuberculous peritonitis in children: Report of two cases and literature review. J. Pediatr. Gastroenterol. Nutr..

[13] Cruz A.T., Starke J.R. (2007). Clinical manifestation of tuberculosis in children. Paediatr. Resp. Rev..

[14] Ridaura-Sanz C., López-Corella E., Lopez-Ridaura R. (2012). Intestinal/peritoneal tuberculosis in children: An analysis of autopsy cases. Tuberc. Res. Treat..

[15] Kedia S., Das P., Madhusudhan K.S., Dattagupta S., Sharma R., Sahni P., Makharia G., Ahuja V. (2019). Differentiating Crohn’s disease from intestinal tuberculosis. World J. Gastroenterol..

[16] Burrill J., Williams C.J., Bain G., Conder G., Hine A.L., Misra R.R. (2007). Tuberculosis: A radiologic review. Radiographics.

[17] Sheikh M., Moosa I., Hussein F.M., Qurttom M.A., Behbehani A.I. (1999). Ultrasonographic diagnosis in abdominal tuberculosis. Australas. Radiol..

[18] Andronikou S., Wieselthaler N. (2004). Modern imaging of tuberculosis in children: Thoracic, central nervous system and abdominal tuberculosis. Pediatr. Radiol..

[19] Engin G., Acunaş B., Acunaş G., Tunaci M. (2000). Imaging of extrapulmonary tuberculosis. Radiographics.

[20] Andronikou S., Welman C.J., Kader E. (2002). The CT features of abdominal tuberculosis in children. Pediatr. Radiol..

[21] Zhao J., Cui M.-Y., Chan T., Mao R., Luo Y., Barua I., Chen M., Li Z.-P., Feng S.-T. (2015). Evaluation of intestinal tuberculosis by multi-slice computed tomography enterography. BMC Infect. Dis..

[22] Nel EDSchaaf H.S., Zumla A.I. (2009). Abdominal Tuberculosis in Children. Tuberculosis: A Comprehensive Clinical Reference.

[23] Saczek K.B., Schaaf H.S., Voss M. (2001). Diagnostic dilemmas in abdominal tuberculosis in children. Pediatr. Surg. Int..

[24] Johnson C.A., Hill I.D., Bowie M.D. (1987). Abdominal tuberculosis in children. A survey of cases at the Red Cross War Memorial Children’s Hospital, 1976–1985. S. Afr. Med. J..

[25] Talwar B.S., Talwar R., Chowdhary B. (2000). Abdominal tuberculosis in children: An Indian experience. J. Trop. Pediatr..

[26] Debi U., Ravisankar V., Prasad K.K., Sinha S.K., Sharma A.K. (2014). Abdominal tuberculosis of the gastrointestinal tract: Revisited. World J. Gastroenterol..

[27] Collins Á.B., Floyd S., Gordon S.V., More S.J. (2022). Prevalence of *Mycobacterium bovis* in milk on dairy cattle farms: An international systematic literature review and meta-analysis. Tuberculosis.

[28] Migliori G.B., Wu S.J., Matteelli A., Zenner D., Goletti D., Ahmedov S., Al-Abri S., Allen D.M., Balcells M.E., Garcia-Basteiro A.L. (2022). Clinical standards for the diagnosis, treatment and prevention of TB infection. Int. J. Tuberc. Lung Dis..

[29] Principi N., Galli L., Lancella L., Tadolini M., Migliori G.B., Villani A., Esposito S., Italian Pediatric TB Study Group (2016). Recommendations Concerning the First-Line Treatment of Children with Tuberculosis. Paediatr. Drugs..

[30] Gan H.T., Chen Y.Q., Ouyang Q., Bu H., Yang X.Y. (2002). Differentiation between intestinal tuberculosis and Crohn’s disease in endoscopic biopsy specimens by polymerase chain reaction. Am. J. Gastroenterol..

[31] Dones P., Di Gangi M., Failla M.C., Genova S., Giannitto C., Corsello G., Principi N., Esposito S. (2014). Intestinal tuberculosis in a child living in a country with a low incidence of tuberculosis: A case report. BMC Res. Notes..

[32] Pecora F., Dal Canto G., Veronese P., Esposito S. (2021). Treatment of Multidrug-Resistant and Extensively Drug-Resistant Tuberculosis in Children: The Role of Bedaquiline and Delamanid. Microorganisms.

[33] Maphalle L.N.F., Michniak-Kohn B.B., Ogunrombi M.O., Adeleke O.A. (2022). Pediatric Tuberculosis Management: A Global Challenge or Breakthrough?. Children.

[34] Bossù G., Autore G., Bernardi L., Buonsenso D., Migliori G.B., Esposito S. (2023). Treatment options for children with multi-drug resistant tuberculosis. Expert Rev. Clin. Pharmacol..

